# Initial Effect of Temperature Rise on α-Synuclein Aggregation – Entropic Forces Drive the Exposure of Protein Hydrophobic Groups Probed by Fluorescence Spectroscopy

**DOI:** 10.1007/s10895-023-03192-5

**Published:** 2023-02-24

**Authors:** Marco A. Saraiva, M. Helena Florêncio

**Affiliations:** 1 Centro de Química Estrutural, Av. Rovisco Pais, Instituto Superior Técnico, University of Lisbon, Campus Alameda, 1049-001 Lisbon, Portugal; 2 Departamento de Química e Bioquímica, Faculdade de Ciências, University of Lisbon, 1749-016 Lisbon, Portugal; 3 Laboratório de FTICR e Espectrometria de Massa Estrutural, Faculdade de Ciências, University of Lisbon, 1749-016 Lisbon, Portugal; 4 Faculdade de Ciências, MARE – Marine and Environmental Sciences Centre / ARNET - Aquatic Research Network, University of Lisbon, 1749-016 Lisbon, Portugal

**Keywords:** α-synuclein, Temperature, Protein aggregation, Protein hydrophobic groups exposure, Fluorescence spectroscopy

## Abstract

The aberrant formation of α-synuclein (Syn) aggregates, varying in size, structure and morphology, has been linked to the development of Parkinson’s disease. In the early stages of Syn aggregation, large protein amyloid aggregates with sizes > 100 nm in hydrodynamic radius have been noticed. These low overall abundant large Syn aggregates are notoriously difficult to study by conventional biophysical methods. Due to the growing importance of studying the early stages of Syn aggregation, we developed a strategy to achieve this purpose, which is the study of the initial effect of the Syn protein aqueous solutions temperature rise. Therefore, the increase of the Syn aqueous solutions entropy by the initial effect of the temperature rise led to the exposure of the protein hydrophobic tyrosyl groups by not interfering with this amyloid protein aggregation. As an attempt to interpret the degree of the referred protein tyrosyl groups exposure, the classic rotameric conformations of the *N*_α_-acetyl-L-tyrosinamide (NAYA) parent compound were used. For both NAYA and Syn, it was determined that the classic rotameric conformations involving the tyrosyl groups indeed accounted for their exposure under steady-state conditions of fluorescence, for lowest molecular species concentrations investigated at least. In this situation, Syn aggregation was observed. For the higher NAYA and Syn concentrations studied, the referred classic rotameric conformation were insufficient in such referred steady-state conditions and, for Syn, in particular, fluorescence anisotropy measurements revealed that less protein aggregation occurs along with its delay. Overall, the developed strategy by focusing on the initial effect of the temperature rise of Syn aqueous solutions in lower concentrations is suitable for informing us about the degree of this protein aggregation in solution.

## Introduction

Parkinson’s disease (PD) is initially characterized by the irreversible damage regarding dopaminergic neurons in the *substancia nigra* of the brain. In PD there are motor symptoms that are exacerbated, including bradykinesia, rigidity and tremor [1]. Post-mortem brain analysis has shown the presence of proteinaceous inclusions, often referred as Lewy bodies, which primarily contain aggregated α-synuclein (Syn) [1]. In terms of the Syn aggregation reaction, the production of intermediate species was an attractive strategy to understand how these referred species evolve from the protein monomers to the putative end stage amyloid fibrils [2, 3]. Such intermediate species, initially designated by Syn oligomers, have been merely lab-generated, often by using high concentrations of the Syn protein [2, 3]. While the mechanism of Syn aggregation has not being completely elucidated, researchers have also been tempted to identify these toxic oligomers species in complex human biofluids and tissues [4]. Very recently, reversible and low abundance of early protein large aggregated species were found to exist in solution. As an example, the formation of low abundant early protein aggregates with sizes > 100 nm in hydrodynamic radius for intrinsically disordered RNA-binding proteins, was reported [5]. These reversible early large aggregated species were formed in subsaturated protein solutions [5]. In another example, using different sucrose gradients and subjecting the Syn solutions to ultracentrifugation, the existence of protein aggregates with lengths > 100 nm was determined and also, when smaller, these large Syn aggregates (lag-phase) were more toxic and they seemed to be, with regard to inflammation and to permeabilization of single-liposome membranes [1]. It appears that a sudden importance is being attributed to early processes governing the aggregation of disordered proteins in solution. Moreover, this more realistic strategy can overpower the classic results of the lab-generated small Syn oligomers, the latter having molecular structures with a few nanometres in gyration radius [6].

In 2020 we observed the early formation of Syn aggregates in solution with a low overall abundance and with sizes > 100 nm in hydrodynamic radius [7]. These large Syn aggregates formation was sensitive to the variation of the solutions conditions, including pH and protein concentration [7, 8]. In our reported studies, the re-suspended Syn stock solution were centrifuged in order to remove high molecular weight protein aggregates by using 100 kDa membrane centrifuge filters [7, 8]. The effect of using different cut-offs of the membrane centrifuge filters, in particular, was investigated by us and, in fact, using the 100 kDa membrane centrifuge filters did remove high molecular weight protein aggregates and, the Syn aggregates present presented a very low overall abundance, as determined ca. 20 min after the centrifugation procedure [9]. The large Syn aggregates formed are not likely to be artefacts of the protein purification procedure but, rather, they are aggregates initially formed in the course of the Syn aggregation reaction [9]. Furthermore, these referred large Syn aggregates were not stable and, primarily dissociated in solution, presenting thus different dissociation profiles due to the solution pH and the protein concentration used [10]. Even the ionic strength of the Syn protein solution and buffer concentration used markedly influence the protein aggregation, as it was determined that the existence of the buffer capacity is important for restoring early Syn aggregation [10]. In this current study, we investigated the effect of varying the initial temperature of the Syn protein solutions and, determined that the entropy in these solutions drive the exposure of protein hydrophobic groups, that could allow semi-quantifying the Syn aggregation. The Syn protein results were compared with those from *N*_α_-acetyl-L-tyrosinamide (NAYA). This latter compound mimetics the peptide bond in proteins and possesses a tyrosine group similarly to those encountered in the Syn molecular structure (the Syn protein has no tryptophan residues).

## Materials and Methods

### Syn Expression and Purification

The pT7-7 plasmid containing the human Syn sequence (kindly provided by Professor Doctor T. Outeiro, IMM, University of Lisbon) was used to overexpress Syn in Escherichia coli BL21 (DE3) bacteria. Syn was purified as previously described [7–13].

### Syn Aggregation Experiments

Syn aggregation experiments were conducted for a protein concentration of 200 µM in 20 mM tris-HCl at pH 6.5 and 37 ºC with constant agitation (650 rpm – eppendorf Thermomixer) for several days [14]. Aliquots of 150 µL were taken from the Syn incubation solution at determined incubation times and diluted in 450 µL of fresh 20 mM tris-HCl buffer at pH 6.5 to give a final volume of 600 µL.

### Spectroscopic Methods

Ultra-violet absorption spectra were measured either in a Beckman DU-70 or in an Agilent Cary 8454 UV–Visible spectrophotometers with 1.0 nm resolution. Steady-state fluorescence emission and excitation spectra were measured using a SPEX Fluorolog 212I spectrofluorimeter. Fluorescence spectra were collected in the S/R mode and in the right angle geometry. Both in absorption and fluorescence, 5 mm path length quartz fused cuvettes were used. The fluorescence quantum yield of Syn (0.029 at 25 °C) was determined by comparison with the quantum yield of NAYA in water (0.047 at 25 °C) [13]. Fluorescence anisotropy measurements were carried out using Glan-Thompson polarizers in the excitation and emission ports of the above referred spectrofluorimeter. The fluorescence anisotropy, <*r*>, of the sample was calculated according to equation:

$$<r> = \frac{{I}_{V,V}-G \times {I}_{V,H}}{{I}_{V,V}+2 \times G \times {I}_{V,H}}$$

where *I*_ex,em_ is the intensity of the emission; *V* and *H* represent the vertical and horizontal alignments, of the excitation and emission polarizers, respectively; and *G* = *I*_H,V_ / *I*_H,H_ is the instrumental correction factor [13].

Fluorescence decays were measured using the time-correlated single photon counting technique as previously described [13].

## Results

The temperature increase is a known trigger of the Syn aggregation [15]. To this purpose, we decided to investigate the effect of increasing the temperature of the Syn solutions, for temperatures ranging from 20 ºC to 80 ºC. The protein solutions were thus maintained in the desired temperatures for 5 min and then the fluorescence spectra were recorded. The aim of these experiments was to study the initial influence that the Syn solution temperature has on this protein aggregation. The rise of the solutions temperature is known to affect the solutions entropy. To this regard, the increase of the solution entropy leads to the increase of molecular interactions and, particularly, in aqueous solutions polar contacts among species are favoured as well and, as expected, the increase of hydrophobic contacts with the rise of the temperature in such solution conditions. In other to prove the latter, we decided to use the NAYA compound, which possesses a hydrophobic tyrosyl group. Beyond this, the NAYA compound mimetics the peptide bond in proteins and with the incorporation in its molecular structure of the referred tyrosyl group becomes a suitable compound for comparison purposes with the Syn protein. Syn possesses four tyrosine residues and no tryptophan residues. Therefore, the effect of the temperature increase in the NAYA aqueous solutions will be first approached and, later on, the results compared with those obtained for the Syn protein, under the same solution conditions.

### Effect of the Initial Temperature Increase of NAYA Aqueous Solutions

**Fig. 1:**
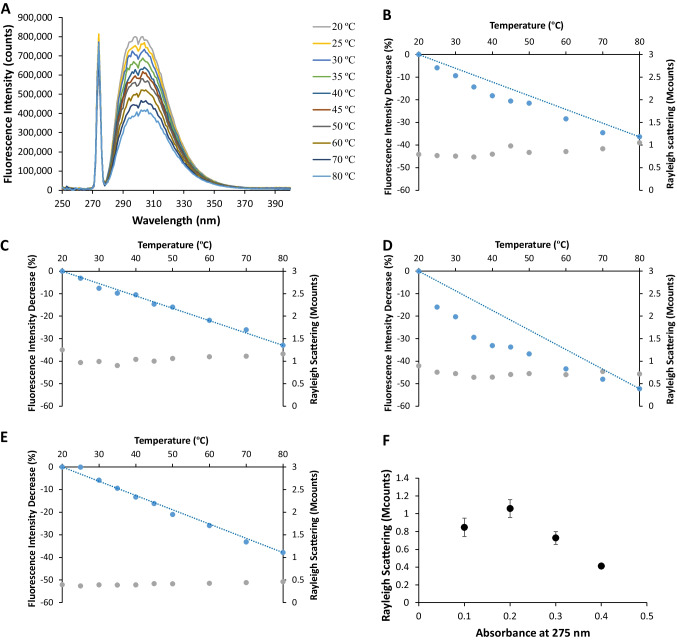
**A** Fluorescence emission spectra for the lowest NAYA concentration studied of 0.67 × 10^− 4^ M (*A*_275nm_ = 0.1; ε = 1490 M^− 1^ cm^− 1^) (10 mM tris-HCl, pH 7) for ten temperature values ranging from 20 ºC to 80 ºC. In the fluorescence spectra the excitation peak at 275 nm can be seen. **B**, **C**, **D** and **E**, the calculated fluorescence intensity decrease (%) (blue points) and the Rayleigh scattering intensity (grey points) for four different NAYA concentrations of 0.67 × 10^− 4^ (*A*_275nm_ = 0.1), 1.3 × 10^− 4^ (*A*_275nm_ = 0.2), 2.0 × 10^− 4^ (*A*_275nm_ = 0.3) and 2.7 × 10^− 4^ M (*A*_275nm_ = 0.4), respectively. For each NAYA concentration, ten temperature values ranging from 20 ºC to 80 ºC were studied. The calculated fluorescence intensity decrease (%) was referenced to the temperature of 20 ºC. Linearity was established between the reference temperature of 20 ºC and the temperature of 80 ºC. **F** Average of Rayleigh scattering values as a function of the absorbance at 275 nm for the NAYA compound (*N* = 10 ± STD)

Within what has been referred above, in Fig. 1a the fluorescence spectra obtained for NAYA aqueous solutions (*A*_275nm_ = 0.1), for temperatures ranging from 20 ºC to 80 ºC, are presented. It becomes clear from this figure that the NAYA fluorescence emission decreases with the increase of the temperature of the NAYA aqueous solutions, as expected. The increase of the solution entropy, due to the temperature increase of the NAYA aqueous solutions, progressively exposes the hydrophobic tyrosyl group to water, and a quenching of its fluorescence emission results. Still considering Fig. 1a, it can be seen that the excitation peak with a maximum at 275 nm did not vary significantly its intensity when increasing the temperature of the NAYA aqueous solutions. This is to be expected, since NAYA is a small compound and, extensive molecular association is not predicted to occur in aqueous solution.

In Fig. 1b and e, we have assembled the results obtained for four NAYA concentrations (*A*_275nm_ = 0.1, 0.2, 0.3 and 0.4) with regard to the calculated fluorescence intensity decrease (%) and to the excitation peak intensity at 275 nm for ten registered temperature values, ranging from 20 ºC to 80 ºC. The referred fluorescence intensity decrease (FID) (%) has been referenced to the temperature of 20 ºC by using the following equation:

$$FID(\%)=\frac{FI_{recorded\;temperature}-FI_{20\;^\circ C}}{FI_{20\;^\circ C}}{\times}100$$

where *FI* represents the collected fluorescence intensity at 305 nm.

**Fig. 2:**
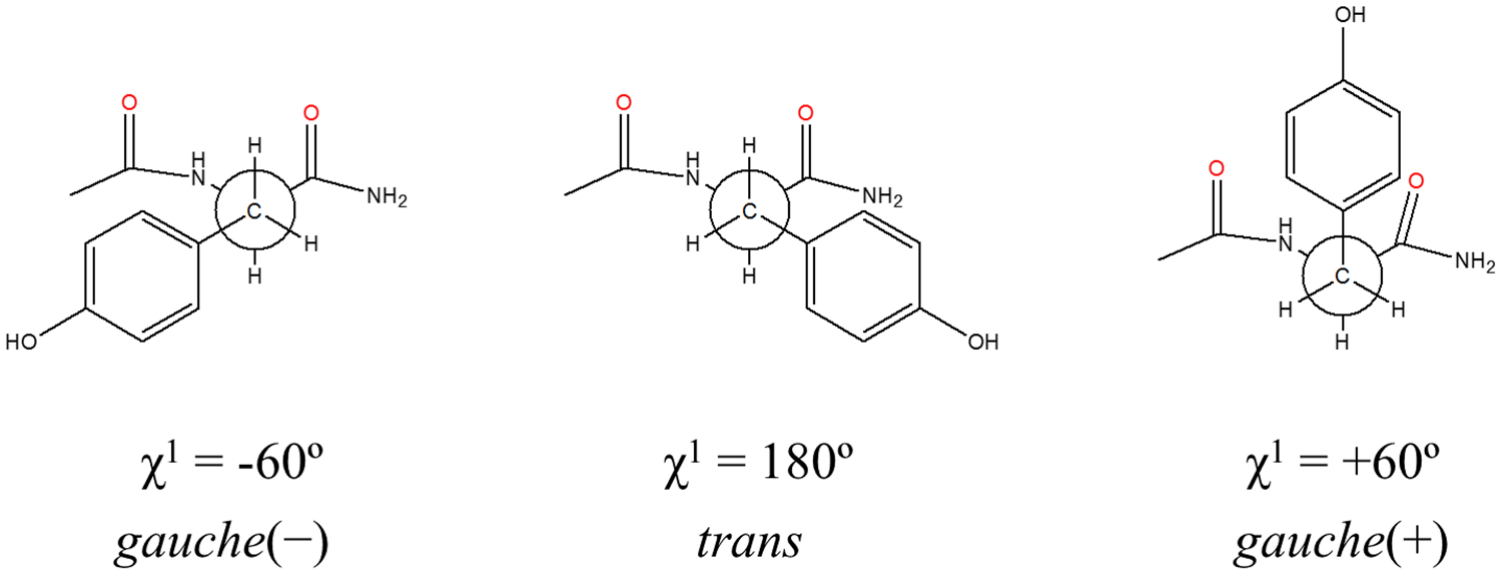
Rotameric conformations of NAYA around C_*α*_ − C_*β*_ bound

In a previous study, we used the classic rotameric conformations of NAYA, in order to interpret the Rayleigh scattering intensity and the fluorescence intensity variations in the NAYA and in the Syn aqueous solutions containing the non-polar solvent 1,4-dioxane [11]. The scenario is not much different with increasing the concentration of NAYA aqueous solutions and subjecting each of the compound concentration to an initial temperature rise, from 20 ºC to 80 ºC. In water, NAYA possesses three rotamers (*gauche*(+), *trans* and *gauche*(−)) (Fig. 2) and, to these, different populations (*gauche*(+): 3–8%, *trans*: 32–34% and *gauche*(−): 60–64%), have been reported [16]. These equilibrium NAYA rotamer populations were determined by NMR [16]. But two situations must be accounted for, in the present study: (i) the exposure of hydrophobic parts of the NAYA molecule should increase with increasing the NAYA concentration in the aqueous solutions and (ii) for a given NAYA concentration in the aqueous solutions the temperature rise should indicate the quenching provided, not only by the presence of surrounding water molecules, but also for polar groups in the NAYA rotamers that provide additional quenching to the tyrosyl groups fluorescence emission. Regarding the above mentioned, the result is therefore the contribution of the quenchers effect in the NAYA tyrosyl groups fluorescence emission. In Fig. 1b, for the lowest NAYA concentration investigated (*A*_275nm_ = 0.1), a negative deviation from linearity can be seen. This retrieves to the referred situation where not only quenching of the NAYA tyrosyl group is provided by the surrounding water molecules but, also, additional quenching is gained by the presence of NAYA polar groups, the carbonyl group in particular, nearby the tyrosyl group in the rotamer conformation with the initial temperature rise of the aqueous solution. In Fig. 1c, for another NAYA concentration studied (*A*_275nm_ = 0.2), no negative deviation from linearity can be seen, which affords for only quenching having been provided by the surrounding water molecules to the NAYA tyrosyl groups fluorescence emission. In this case, NAYA polar groups (carbonyl) should not be adjacent to the tyrosyl group in the rotamer conformation, with the temperature rise of the NAYA aqueous solution. In Fig. 1d, for an increased NAYA concentration (*A*_275nm_ = 0.3), a more negative deviation from linearity can be seen, in comparison to that observed in Fig. 1b. In Fig. 1e, for the higher NAYA concentration (*A*_275nm_ = 0.4) investigated, again no negative deviation can be seen from linearity, which affords for the fact that only quenching is being provided by the surrounding water molecules to the NAYA tyrosyl groups fluorescence emission, as similarly described in Fig. 1c. Attending to the classic rotameric conformations in Fig. 2, the NAYA amide carbonyl in the *trans* rotamer, which represents a carbonyl with a less negative charge density and adjacent to NAYA tyrosyl group, can provide minor quenching in the tyrosyl group fluorescence emission. Moreover, the NAYA acetyl carbonyl in the *gauche*(−) rotamer, which represents a carbonyl with a more negative charge density and adjacent to NAYA tyrosyl group, can provide higher quenching in the tyrosyl group fluorescence emission. Also, in the *gauche*(+) rotamer, in which both NAYA amide carbonyl and NAYA acetyl carbonyl can be far apart from the NAYA tyrosyl group, specifically in a closed shape conformation due to the hydrophobic character of the rotamer exposed due to the initial temperature rise, prevents additional quenching in the tyrosyl group fluorescence emission. Therefore, from Fig. 1b and d, the population of the NAYA rotamers varies, which depends on the rotamers interconversion rates, being the most populated the *trans*, *gauche*(+) and *gauche*(−) rotamers, respectively. It should be borne in mind that with increasing the NAYA concentration (and with the temperature rise) we are dealing with more solvent exposed hydrophobic NAYA molecular species, and that should affect the NAYA rotamers populations, in comparison to those latter in aqueous solutions. Another question to be answered is which of the three NAYA rotamers referred is most populated for the higher NAYA concentration investigated (*A*_275nm_ = 0.4). In Fig. 1f, the Rayleigh scattering variation for the NAYA compound is shown as a function of its absorbance at 275 nm. Although, the Rayleigh scattering values do not vary significantly with the initial temperature rise (Fig. 1b and e), they are inferior with respect to the higher NAYA concentration investigated (Fig. 1f). Meaning that, for this higher NAYA concentration, the NAYA molecular species scatters light less efficiently. We, therefore, anticipate that for the higher NAYA concentration, the increase of intermolecular interactions among NAYA hydrophobic groups possibly disturb more structured NAYA rotameric conformations and, the likely result is NAYA adopting a more extended conformation. The latter polar groups, such as carbonyls, in the NAYA molecular structure should not provide additional quenching to the NAYA tyrosyl groups fluorescence emission.

**Fig. 3:**
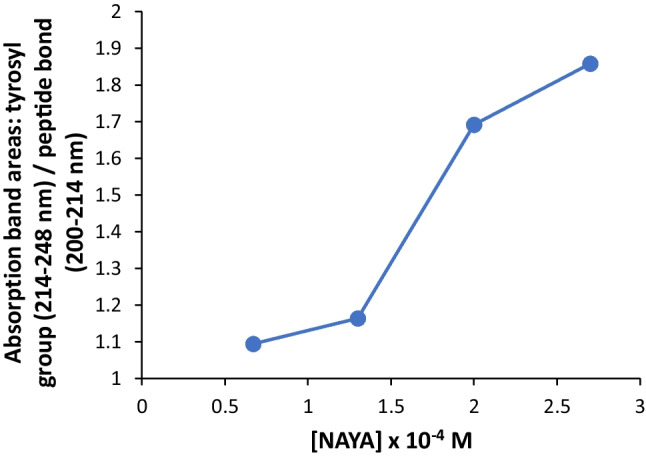
Absorption band area of the NAYA tyrosyl group (214–248 nm) divided by the absorption band area of the NAYA peptide bond (200–214 nm) as a function of the NAYA concentration. The absorption spectra were determined at 25 ºC (10 mM tris-HCl, pH 7). The NAYA concentrations here used are the same as those refereed in Fig. 1

We referred above that with increasing the NAYA concentration (and with the temperature rise) in aqueous solution, there is a progressive exposure of NAYA hydrophobic groups due the increase of the solution entropy. We further determined the absorption spectra for NAYA aqueous solutions at 25 ºC and calculated the far UV absorption band areas corresponding to the tyrosyl group with a maximum at 223 nm and to the peptide bond (Fig. 3). This retrieves for the influence that the NAYA tyrosyl group has on the absorption of the NAYA peptide bond and, more specifically, the likely exposure of the NAYA hydrophobic tyrosyl group in aqueous solution due to the increase of the solution entropy. In Fig. 3, it can be seen that minor exposure of the NAYA tyrosyl group occurs for NAYA concentrations of 0.67 × 10^− 4^ (*A*_275nm_ = 0.1) to 1.3 × 10^− 4^ (*A*_275nm_ = 0.2). Furthermore, for NAYA concentrations of 1.3 × 10^− 4^ (*A*_275nm_ = 0.2) to 2.0 × 10^− 4^ (*A*_275nm_ = 0.3) increased exposure of the NAYA hydrophobic tyrosyl group is observed (Fig. 3). Finally, for NAYA concentrations of 2.0 × 10^− 4^ (*A*_275nm_ = 0.3) to 2.7 × 10^− 4^ (*A*_275nm_ = 0.4) minor exposure of the NAYA hydrophobic tyrosyl group occurs again (Fig. 3). These results are in agreement with those depicted in Fig. 1, where additional NAYA tyrosyl groups fluorescence emission quenching is observed (increased exposure of NAYA tyrosyl groups) for NAYA concentrations of 1.3 × 10^− 4^ (*A*_275nm_ = 0.2) to 2.0 × 10^− 4^ (*A*_275nm_ = 0.3). For NAYA concentrations of 0.67 × 10^− 4^ (*A*_275nm_ = 0.1) to 1.3 × 10^− 4^ (*A*_275nm_ = 0.2) and, also for NAYA concentrations of 2.0 × 10^− 4^ (*A*_275nm_ = 0.3) to 2.7 × 10^− 4^ (*A*_275nm_ = 0.4), additional quenching of the NAYA hydrophobic tyrosyl groups fluorescence emission is minor or not observed (decreased exposure of NAYA tyrosyl groups).

### Effect of the Initial Temperature Increase of Syn Aqueous Solutions

**Fig. 4:**
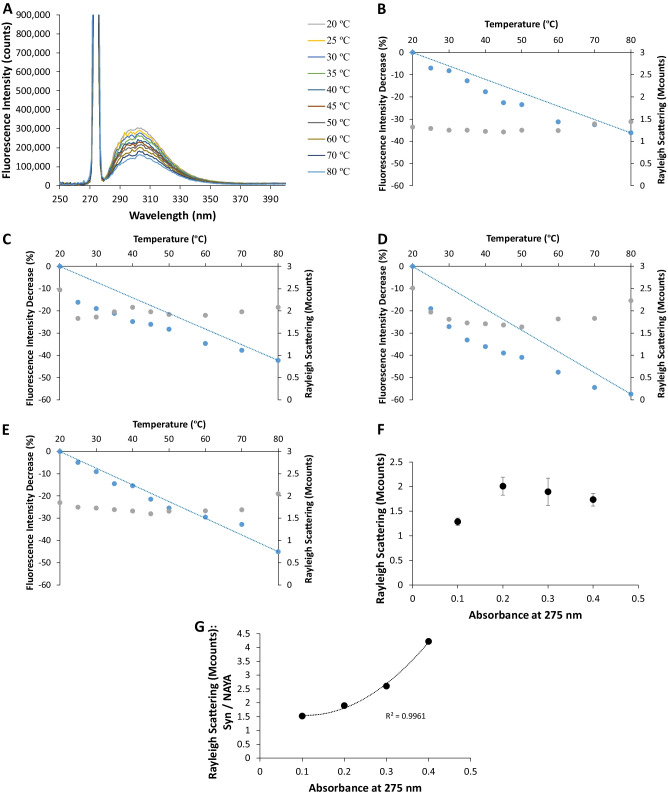
**A** Fluorescence emission spectra for the lowest Syn protein concentration studied of 16.7 µM (*A*_275nm_ = 0.1; ε = 5974 M^− 1^ cm^− 1^) (10 mM tris-HCl, pH 7) for ten temperature values ranging from 20 ºC to 80 ºC. In the fluorescence spectra the partial excitation peak at 275 nm. **B**, **C**, **D** and **E** can be seen, together with the calculated fluorescence intensity decrease (%) (blue points) and the Rayleigh scattering intensity (grey points) for four different Syn protein concentrations of 16.7 (*A*_275nm_ = 0.1), 33.5 (*A*_275nm_ = 0.2), 50.2 (*A*_275nm_ = 0.3) and 67.0 µM (*A*_275nm_ = 0.4), respectively. For each Syn protein concentration, ten temperature values ranging from 20 ºC to 80 ºC were studied. The calculated fluorescence intensity decrease (%) was referenced to the temperature of 20 ºC. Linearity was established between the reference temperature of 20 ºC and the temperature of 80 ºC. **F** Average of Rayleigh scattering values as a function of the absorbance at 275 nm for the Syn protein (*N* = 10 ± STD). **G** Average of Rayleigh scattering values of Syn divided by the average of Rayleigh scattering values for NAYA as a function of the absorbance at 275 nm. In this latter representation, a polynomial line was fitted to the data points

**Fig. 5:**
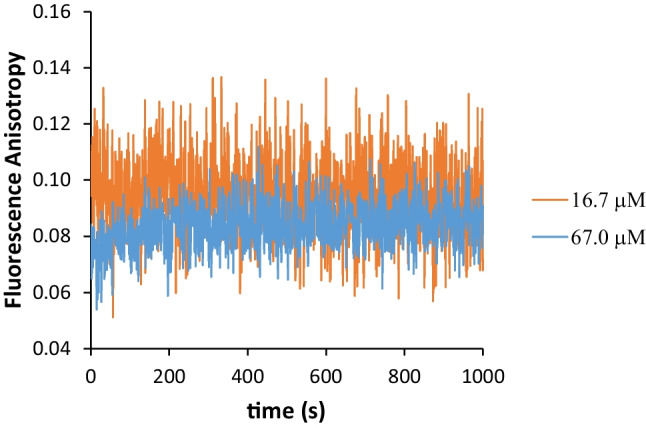
Fluorescence anisotropy of Syn at two different concentrations, of 16.7 and 67.0 µM (10 mM tris-HCl, pH 7 and 25 ºC), as a function of time (first 1000 s)

Syn is a small amyloid protein with 14.46 kDa and 140 amino acid residues. It possesses four tyrosine residues and its propention to aggregate makes it suitable to be studied by intrinsic fluorescence spectroscopy and by Rayleigh scattering determination. In Fig. 4a, the fluorescence emission spectra for the Syn protein with a concentration of 16.7 µM (*A*_275nm_ = 0.1) is presented and, with varying the solution temperature, for temperatures ranging from 20 ºC to 80 ºC. Similarly to the fluorescence spectra presented in Fig. 1a, i.e. for the NAYA compound, the Syn fluorescence emission decreases with the increase of the solution temperature, as expected (Fig. 4a). The excitation peak at 275 nm for Syn is of much higher intensity (Fig. 4a) than the one observed for the NAYA compound (Fig. 1a), which is also expected due to the size of Syn protein and to its propention to aggregate in solution. In Fig. 4b and e, we have assembled the results obtained from the fluorescence emission spectra for the Syn protein, which includes the investigation of four protein concentrations. Moreover, in each of them, ten temperatures were studied, ranging from 20 ºC to 80 ºC. In Fig. 4b and d, in the context of the classic rotameric conformations earlier referred for the Syn protein, a negative deviation from linearity for the calculated fluorescence intensity decrease (%) for the NAYA compound, mainly occurs. Accordingly to what was discussed for NAYA, *gauche*(−) and *trans* rotamers are the molecular configurations that develop negative deviation from linearity for the calculated fluorescence intensity decrease (%). But attending to the fact that only peptide bonds (amide formation) and not the acetyl group are considered to exist in the Syn molecular structure, we can assume that the negative deviation from linearity for the calculated fluorescence intensity decrease (%), in Fig. 4b and d, is due to the existence of the *trans* rotamer. Considering the *trans* rotameric conformation is being the most populated with increasing the Syn protein concentration in aqueous solution (and with the temperature rise), we can infer that the increase of the negative deviation from linearity for the calculated fluorescence intensity decrease (%) can signify, in fact, an increase of hydrophobic groups formation in the Syn molecular structure. This latter can be related with increased Syn protein aggregation as the protein concentration increases in the aqueous solution. In Fig. 4f, the Rayleigh scattering variation for Syn as a function of its absorbance at 275 nm is presented. Although, the Rayleigh scattering values do not vary significantly with the initial temperature rise (Fig. 4b and e), they are higher for the Syn protein (Fig. 4f), in comparison to those for the NAYA parent compound (Fig. 1f), as expected. This is due to the Syn protein aggregation. Nevertheless, these latter Rayleigh scattering values determined for the four Syn protein concentrations follow roughly the same trend (Fig. 4f), when compared to those Rayleigh scattering values for the four NAYA concentrations studied (Fig. 1f). Within the above mentioned, i.e. concerning the Rayleigh scattering values variation, it is clear that Syn protein aggregation is occurring (Fig. 4g). Accordingly to what has been above referred, the *trans* rotameric configuration is the most populated with increasing the Syn concentration (and with the temperature rise) and a negative deviation from linearity is observed for the calculated fluorescence intensity decrease (%) in such solution conditions (Fig. 4b and d). We were puzzled with the result obtained in Fig. 4e, for the higher Syn protein concentration investigated. In the latter, no deviation from linearity for the calculated fluorescence intensity decrease (%) with the initial temperature rise can be observed. We, therefore, anticipate that the interpretation provided by the use of the classic rotameric conformations is insufficient to elucidate the phenomena related to the Syn protein aggregation with increasing the Syn protein concentration (and with the temperature rise). In order to circumvent this aspect, we decided to determine the fluorescence anisotropy for the Syn solutions. In Fig. 5 the fluorescence anisotropy (< *r*>) for two Syn concentrations, of 16.7 and 67.0 µM (10 mM tris-HCl, pH 7 and 25 ºC) is presented. It is evident from this figure that higher rotational mobility of Syn occurs for the higher Syn concentration (< *r* > = 0.08 ± 0.01), in comparison to the lower Syn concentration (< *r* > = 0.10 ± 0.01). This indicates that increased Syn protein aggregation likely occurs for the lower Syn concentration (16.7 µM), in comparison to the higher Syn concentration studied (67.0 µM). In fact, from Fig. 5a slight increase of the fluorescence anisotropy for the higher Syn concentration (67.0 µM) can be seen in the first 200 s, which can be due to the predicted enhanced Syn aggregates dissociation occurring at this protein concentration [10, 12].

**Fig. 6:**
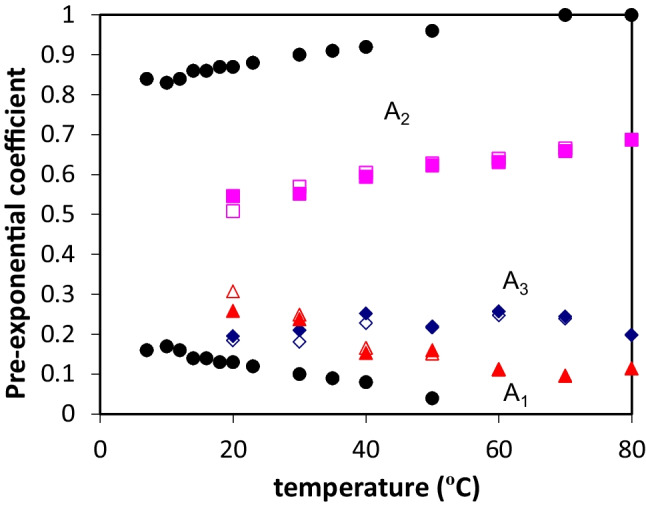
Fluorescence pre-exponential coefficients for a 67.0 µM protein concentration (10 mM tris-HCl, pH 7) as a function of temperature with λ_exc_ = 278 nm and λ_em_ = 295 nm. For Syn, close and open symbols refer to the heating and cooling of protein solutions, respectively. For comparison, data for NAYA (black symbols) are also shown

We also thought about a methodology that could be implemented in order to confirm our hypothesis on the highest protein concentration investigated (67.0 µM), concerning the fact that protein aggregation is indeed occurring at that concentration and also that the protein tyrosines *trans* molecular rotamer predominates when the protein solutions temperature increase. Therefore, we performed time-resolved fluorescence spectroscopy (TRFS) measurements on a 67.0 µM protein concentration (*A*_275 nm_ = 0.4) (10 mM tris-HCl, pH 7) with varying the protein solution temperature from 20 ºC to 80 ºC (Fig. 6). The fluorescence decays obtained required sums of three exponentials to be perfectly fitted. Consequently, in each TRFS measurement three decay times along with three pre-exponential coefficients were obtained. The calculated decay times (τ) which do not vary when the temperature increases from 20 ºC to 80 ºC, followed the order: τ_1_ ≈ 1.7 ns > τ_2_ ≈ 0.7 ns > τ_3_ ≈ 0.15 ns. Importantly, the corresponding three pre-exponential coefficients varied with the protein solution temperature increase (Fig. 6). In the case of NAYA, double exponential decays in water were previously rationalized on the basis of the three rotamers model, being only two of these significantly populated [17], while in tri-peptides [16] and proteins [18] all three rotamers are populated, leading to triple exponential decays. Thus, considering the current study and on the basis of three rotamers model for NAYA (10 mM tris-HCl, pH 7) the *gauche*(+) is the long-lived rotamer, followed by the quenched *trans* rotamer and finally by the highest quenched and short-lived *gauche*(−) rotamer (Fig. 1b and d). Accordingly, with the equilibrium rotamer populations determined by NMR [16] the NAYA *gauche*(−) rotamer is the most populated, followed by the NAYA *trans* rotamer and finally by the less populated NAYA *gauche*(+) rotamer. Nevertheless, for large peptides (and proteins) the *trans* rotamer is the most populated, followed by the *gauche*(−) rotamer and finally the *gauche*(+) rotamer is still the less populated [16]. The above order for the rotamers populations in indeed fairly observed in Fig. 6, in which the Syn tyrosines *trans* rotamer is the most populated (A_2_), followed by the less populated Syn tyrosines *gauche*(−) (A_3_) and Syn tyrosines *gauche*(+) (A_1_) rotamers. While the Syn tyrosines *gauche*(−) rotamer population (A_3_) does not significantly vary with the protein solutions temperature increase, the population of the long-lived Syn tyrosines *gauche*(+) rotamer (A_1_) seems to decrease when increasing the temperature of the Syn solutions. Since the long-lived Syn tyrosines *gauche*(+) rotamer reports mainly to water exposure, the fact that its population decreases with the temperature increase of the Syn solutions, indicates that the protein tyrosines are, in fact, sensing a less hydrophilic environment. To this regard, protein aggregation is indeed occurring with the increase of the protein solutions temperature. Also, the results from steady-state fluorescence corroborate the TRFS results, of which the protein tyrosines *trans* rotamer is being the most populated rotamer and, therefore, can report for the conformation alterations occurring in the Syn protein while the temperature of its solutions is increased.

**Fig. 7:**
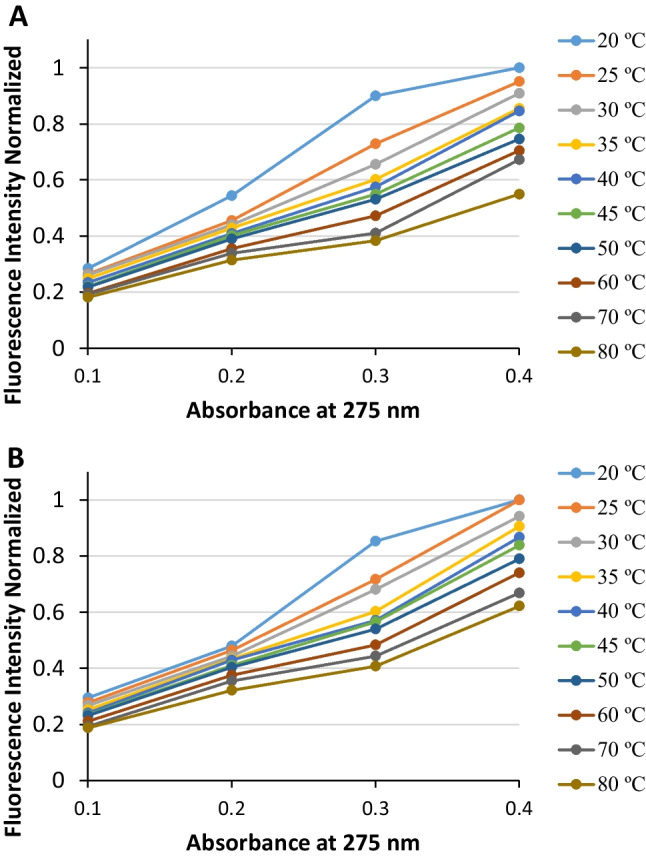
**A** Normalized fluorescence intensity of Syn (obtained for each temperature, of 20 ºC to 80 ºC) as a function of the absorbance at 275 nm. **B** Normalized fluorescence intensity of NAYA (obtained for each temperature, of 20 ºC to 80 ºC) as a function of the absorbance at 275 nm

One question that remains to be answered concerns whether the initial temperature rise of Syn solutions is triggering the Syn protein aggregation (along with its increase of the protein concentration). From Fig. 7a, b, it can be seen for both the NAYA parent compound and the Syn protein that the fluorescence intensity normalized values trend lines are similar for the temperatures studied, from 20 ºC to 80 ºC. In spite of this, a slight increase of the fluorescence intensity normalized data points scattering is observed for Syn, in comparison to NAYA, i.e. with respect to the variation of the species concentration (Fig. 7a, b). This aspect can retrieve that the Syn aggregation is being triggered by the increase of the protein concentration and not by the initial temperature rise of the protein solutions. In fact, the initial temperature rise of the Syn protein solutions merely confers an increase of the Syn solutions entropy with a likely exposure of the protein species hydrophobic groups. Overall, we can assume that the initial temperature rise of the Syn protein solutions can be a suitable method for studying this protein aggregation by not interfering with it.

### Accessing if the Syn Protein Tyrosines Trans Conformer Structure is Also Relevant in Fibrils

**Fig. 8:**
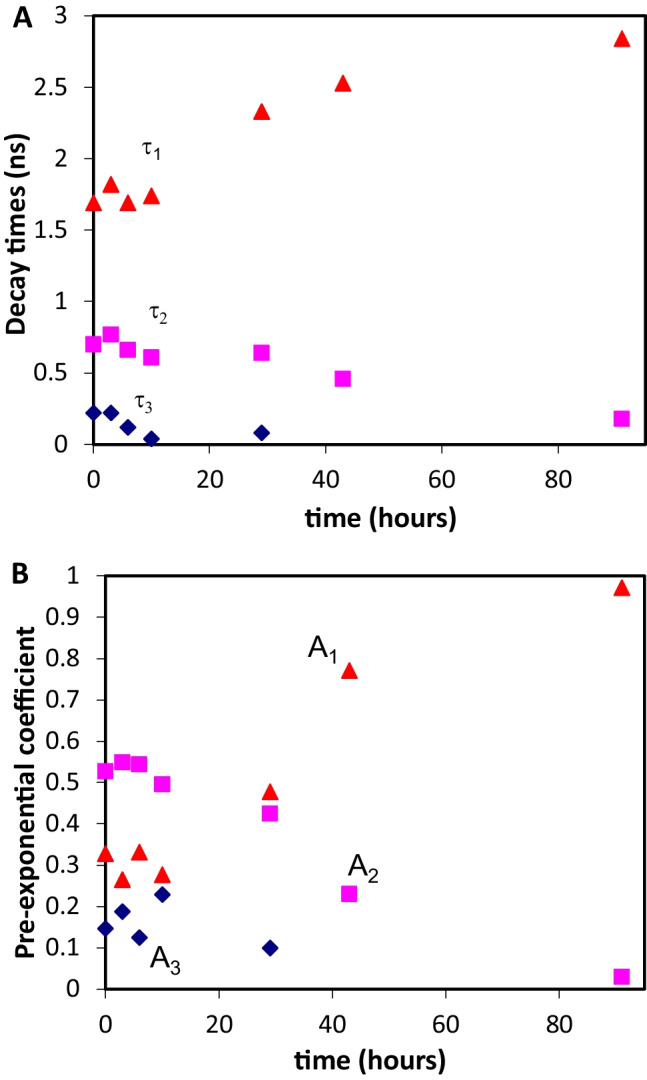
Fluorescence decay times (**A**) and pre-exponential coefficients (**B**) determined for aliquots of an incubated Syn solution at 37 ºC with a 200 µM protein concentration (20 mM tris-HCl, pH 6.5) with λ_exc_ = 278 nm and λ_em_ = 295 nm

In order to determine if the Syn tyrosines *trans* conformer structure is also relevant in fibrils, we decide to incubate Syn protein solutions with a 200 µM concentration (20 mM tris-HCl, pH 6.5) under constant agitation (650 rpm) for 91 h (ca. 3.8 days) at 37 ºC [14]. We did not perform the thioflavin T assay in order to monitor the Syn aggregation reaction leading to the formation of fibrils, but we used identical experimental conditions as the ones elsewhere reported and the authors determined that the Syn aggregation reaction reached a plateau at ca. 40 h [14]. In Fig. 8, the TRFS measurements of aliquots taken of the above referred incubation are shown. The decay times varied with the time of incubation, especially for the more long-lived ones (τ_1_ and τ_2_) (Fig. 8a). Moreover, their corresponding pre-exponential coefficients (A_1_ and A_2_) varied with the time of incubation (Fig. 8b). Surprisingly, we observed that the pre-exponential coefficient A_2_, corresponding to the Syn protein tyrosines *trans* rotamer, decreased with the time of the protein aggregation reaction (Fig. 8b). Also, the pre-exponential coefficient A_1_, corresponding to the Syn protein tyrosines *gauche*(+) rotamer, generally increased with the time of the protein aggregation reaction (Fig. 8b). From the observations above referred, two scenarios can be depicted. Firstly, while the Syn protein tyrosines *trans* rotamer appeared to be important in early Syn aggregation (Fig. 6) it does not seem to be relevant in late Syn aggregation where fibrils are formed (Fig. 8). Secondly, the Syn protein tyrosines *gauche*(+) rotamer which reports to water exposure, as already mentioned, indicates that the Syn protein tyrosines are progressively sensing a more hydrophilic environment with time of the protein aggregation. This essentially means that in the fibrils molecular structure the protein tyrosine residues are not likely to be a part of the fibrils hydrophobic core. Nevertheless, the TRFS measurements, in terms of the obtained pre-exponential coefficients, clearly indicate that the hydrophobic protein tyrosine residues have different roles in the early and in the late protein aggregation reaction where it is likely that these residues account for a different organization of the hydrophobic cores developed in the corresponding aggregates molecular structures.

## Discussion

In this report we intend to determine the effect of varying the temperature of, the NAYA compound and the Syn protein, for different concentrations of each molecular species in buffered solutions (10 mM tris-HCl, pH 7). Both NAYA and the Syn protein have hydrophobic groups that can suffer exposure, while increasing the entropy due to the initial temperature rise of their aqueous solutions. Monitoring the initial influence of the temperature rise in solution, can be a suitable strategy not only for predicting the hydrophobic groups solvent accessibility but also to determine, in the case of the Syn protein, the extent of its aggregation in solution by not interfering with it. Since both NAYA and the Syn protein possess tyrosyl groups, intrinsic fluorescence spectroscopy can be used to probe tyrosyl groups conformational changes. This, in combination with the Rayleigh scattering determination, gives information on the level of association in solution that can be accomplished. We, therefore, decided to firstly use the classic rotameric conformations of the tyrosyl group [16], in order to interpret the effect of increasing the NAYA compound concentration in solution (and the temperature rise). Meaning that, we were searching for a molecularly conformational model that could eventually interpret the conformational changes arising when hydrophobic tyrosyl groups are exposed to the initial temperature rise of the aqueous solutions. For the NAYA compound, and considering its lowest concentrations investigated, we determined that the referred rotameric conformations that provide additional fluorescence emission quenching of the tyrosyl groups are: the *trans* and the *gauche*(−) rotamers, being the latter the most effective into that purpose. The determination of absorption spectra for NAYA at 25 ºC, under the same solution conditions, and the calculated far UV absorption bands areas retrieved similar exposure of NAYA tyrosyl groups, as found in terms of fluorescence spectroscopy measurements. For the Syn protein and also considering its lowest concentrations studied, we inferred that the mentioned rotameric conformations that provide additional fluorescence emission quenching of the tyrosyl groups is the *trans* rotamer. Moreover, the population increase of the *trans* rotamer in the Syn protein solutions with increasing the protein concentration (and the temperature rise) was noticed and can be related to the increase of this amyloid protein aggregation in such solution conditions. For the highest NAYA and Syn concentration studied, we were puzzled. For NAYA, we anticipate that for the higher NAYA concentration the increase of intermolecular interactions among NAYA tyrosyl groups possibly disturb more structured NAYA rotameric conformations and the likely result is NAYA adopting a more extended conformation. In the latter, polar groups, such as carbonyls, in the NAYA molecular structure should not provide additional quenching to the NAYA tyrosyl groups fluorescence emission. For Syn, we therefore assumed that the interpretation provided by the use of the rotameric conformations is insufficient to elucidate the phenomena related to the Syn protein aggregation with further increase of the Syn protein concentration (and with the temperature rise). In order to circumvent this aspect, we decided to perform TRFS measurements and we have therefore determined that the Syn tyrosines *trans* rotamer is the most populated in the Syn solutions alongside that protein aggregation is indeed occurring at the highest Syn concentration investigated (67.0 µM). We also decided to determine the fluorescence anisotropy for the Syn solutions. In fact, fluorescence anisotropy measurements revealed that increased Syn protein aggregation likely occurs for the lower Syn concentration (16.7 µM), in comparison to the higher Syn concentration studied (67.0 µM), and Syn aggregation becomes therefore delayed. Additionally, the initial slight increase of the fluorescence anisotropy for the higher Syn protein concentration investigated (67.0 µM) led to the assumption that enhanced Syn aggregates dissociation is occurring at this protein concentration, which corroborates the above referred that is, for higher Syn concentrations, protein aggregation is indeed delayed.

## Conclusion

Herein, we investigated the initial influence that the temperature rise has on the Syn protein aqueous solutions. The referred initial temperature rise of Syn solutions was determined not to interfere with its aggregation. By comparison with the NAYA parent compound, it was retrieved that such a temperature rise of the Syn aqueous solutions led to the exposure of protein tyrosyl groups. These latter exposed tyrosyl groups can be semi-quantified in order to determine the degree of the Syn protein aggregation in solution. Moreover, the classic rotameric conformations involving the tyrosyl group are suitable for interpreting the exposure of tyrosyl groups with the initial temperature rise of the NAYA parent compound and of the Syn protein solutions, particularly for the lowest concentrations here studied. For the highest concentration investigated in this study, the classic rotameric conformational model involving the tyrosyl group is insufficient under steady-state conditions of fluorescence at least, for both the NAYA parent compound and the Syn protein. In order to circumvent this aspect, we performed TRFS measurements and determined that Syn aggregation still occurs for the highest protein concentration investigated (67.0 µM). We also used fluorescence anisotropy and for the Syn protein, in particular, it was once more confirmed the already reported results that is, that for higher Syn protein concentrations aggregation is delayed. Moreover, for higher Syn protein concentrations, protein aggregates dissociation is enhanced. In sum, by exposing amyloid species, hydrophobic groups with the initial temperature rise of aqueous solutions, we can predict the degree of aggregation of amyloid species in solution by not interfering with it.

## Data Availability

All data analysed during this work are included in the submitted manuscript.
